# Bis[1,3-bis­(1-ethyl-1*H*-benzimidazol-2-yl)-2-oxapropane]­cadmium(II) dipicrate dimethyl­formamide disolvate

**DOI:** 10.1107/S1600536811018228

**Published:** 2011-05-20

**Authors:** Huilu Wu, Bin Liu, Fan Kou, Fei Jia, Jin Kong

**Affiliations:** a School of Chemical and Biological Engineering, Lanzhou Jiaotong University, Lanzhou 730070, People’s Republic of China

## Abstract

In the title compound, [Cd(C_20_H_22_N_4_O)_2_](C_6_H_2_N_3_O_7_)_2_·2C_3_H_7_NO, the Cd^II^ ion is coordinated by four N atoms and two O atoms from two tridentate 1,3-bis­(1-ethyl-1*H*-benzimid­azol-2-yl)-2-oxapropane ligands in a distorted octa­hedral environment.

## Related literature

For related structures with bis­(2-benzimidazol­yl)alkanes and their derivatives, see: Addison *et al.* (1983 ▶); Cheng *et al.* (2004 ▶); Wu *et al.* (2009*a* ▶,*b* ▶); Yun *et al.* (2008 ▶).
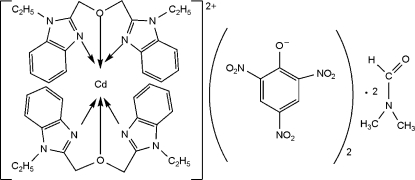


## Experimental

### 

#### Crystal data

[Cd(C_20_H_22_N_4_O)_2_](C_6_H_2_N_3_O_7_)_2_·2C_3_H_7_NO
*M* *_r_* = 1383.65
Triclinic, 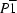

*a* = 14.1182 (3) Å
*b* = 14.3444 (3) Å
*c* = 16.0971 (4) Å
α = 98.287 (1)°
β = 107.969 (1)°
γ = 95.526 (1)°
*V* = 3034.05 (12) Å^3^
*Z* = 2
Mo *K*α radiation
μ = 0.45 mm^−1^
*T* = 153 K
0.33 × 0.25 × 0.14 mm

#### Data collection

Rigaku R-AXIS Spider diffractometer
Absorption correction: multi-scan (*ABSCOR*; Higashi 1995 ▶) *T*_min_ = 0.867, *T*_max_ = 0.940
29100 measured reflections
13317 independent reflections
11890 reflections with *I* > 2σ(*I*)
*R*_int_ = 0.019

#### Refinement

*R*[*F* ^2^ > 2σ(*F* ^2^)] = 0.028
*wR*(*F* ^2^) = 0.085
*S* = 1.10
13317 reflections
839 parameters
H-atom parameters constrained
Δρ_max_ = 0.64 e Å^−3^
Δρ_min_ = −0.58 e Å^−3^

### 

Data collection: *RAPID-AUTO* (Rigaku/MSC, 2004) ▶; cell refinement: *RAPID-AUTO* ▶; data reduction: *RAPID-AUTO* ▶; program(s) used to solve structure: *SHELXS97* (Sheldrick, 2008 ▶); program(s) used to refine structure: *SHELXL97* (Sheldrick, 2008 ▶); molecular graphics: *SHELXTL* (Sheldrick, 2008 ▶); software used to prepare material for publication: *SHELXTL*.

## Supplementary Material

Crystal structure: contains datablocks global, I. DOI: 10.1107/S1600536811018228/rn2085sup1.cif

Structure factors: contains datablocks I. DOI: 10.1107/S1600536811018228/rn2085Isup2.hkl

Additional supplementary materials:  crystallographic information; 3D view; checkCIF report

## Figures and Tables

**Fig. 1.**
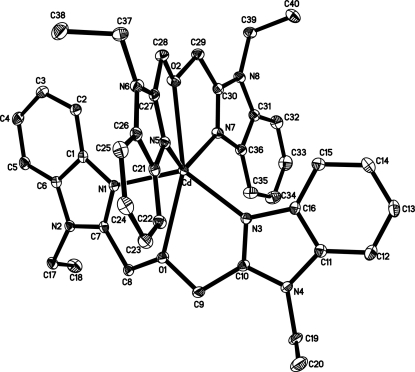
Molecular structure and atom numbering for the cation. Hydrogen atoms have been omitted for clarity and the displacement ellipsoids are shown at the 30% probability level.

## supplementary crystallographic information

### Comment

Interest in bis(2-benzimidazolyl)alkanes and their derivatives is widespread (Addison *et al.*, 1983; Cheng *et al.*, 2004). We have previously reported the crystal structure of some related complexes (Wu *et al.*, 2009*a*,*b*; Yun *et al.*, 2008). The asymmetric unit of the title compound consists of a discrete di[1,3-bis(1-ethyl-1*H*-benzimidazol-2-yl)-2-oxapropane] cadmium(II) cation, two picrate anions and two molecules of dimethylformamide. The cadmium ion is six-coordinate with a N_4_O_2_ ligand set. The etobb ligand acts as a tridentate N-donor and O-donor. The coordination geometry of the Cd^II^ may be best described as distorted octahedral with four coordination nitrogen atoms from an ideal equatorial plane. The maximum deviation (N7) from the plane containing these four N atoms is 1.087 (2) Å. The axial sites are occupied by O1 and O2. The O atoms can be considered as weakly coordinated.

### Experimental

To a stirred solution of 1,3-bis(1-ethyl-1*H*-benzimidazol-2-yl)-2-oxapropane (0.167 g, 0.5 mmol) in hot MeOH (15 ml) was added Cd(C_6_H_2_N_3_O_7_)_2_ (0.153 g, 0.25 mmol) in MeOH (5 ml). A yellow crystalline product formed rapidly. The precipitate was filtered off, washed with MeOH and absolute Et_2_O, and dried *in vacuo*. The dried precipitate was dissolved in DMF resulting in a yellow solution. The yellow crystals suitable for X-ray diffraction studies were obtained by ether diffusion into DMF after three days at room temperature. Yield, 0.189 g (59%). (found: C, 50.21; H, 4.41; N,16.32. Calcd. for C_58_H_62_CdN_16_O_18_: C, 50.35; H, 4.52; N, 16.20)

### Refinement

All H atoms were found in difference electron maps and were subsequently refined in a riding-model approximation with C—H distances ranging from 0.95 to 0.99 Å and *U*_iso_(H) = 1.2 *U*_eq_ or *U*_iso_(H) = 1.5 *U*_eq_ of the carrier atom, respectively.

### Crystal data

**Table:**

[Cd(C_20_H_22_N_4_O)_2_](C_6_H_2_N_3_O_7_)_2_·2C_3_H_7_NO	*Z* = 2
*M**_r_* = 1383.65	*F*(000) = 1428
Triclinic, *P*1	*D*_x_ = 1.515 Mg m^−^^3^
*a* = 14.1182 (3) Å	Mo *K*α radiation, λ = 0.71073 Å
*b* = 14.3444 (3) Å	Cell parameters from 7749 reflections
*c* = 16.0971 (4) Å	θ = 3.2–27.5°
α = 98.287 (1)°	µ = 0.45 mm^−^^1^
β = 107.969 (1)°	*T* = 153 K
γ = 95.526 (1)°	Block, yellow
*V* = 3034.05 (12) Å^3^	0.33 × 0.25 × 0.14 mm

### Data collection

**Table:**

Rigaku R-AXIS Spider diffractometer	13317 independent reflections
Radiation source: fine-focus sealed tube	11890 reflections with *I* > 2σ(*I*)
graphite	*R*_int_ = 0.019
φ and ω scans	θ_max_ = 27.5°, θ_min_ = 3.0°
Absorption correction: multi-scan (*ABSCOR*; Higashi 1995)	*h* = −16→18
*T*_min_ = 0.867, *T*_max_ = 0.940	*k* = −18→18
29100 measured reflections	*l* = −20→20

### Refinement

**Table:**

Refinement on *F*^2^	Secondary atom site location: difference Fourier map
Least-squares matrix: full	Hydrogen site location: inferred from neighbouring sites
*R*[*F*^2^ > 2σ(*F*^2^)] = 0.028	H-atom parameters constrained
*wR*(*F*^2^) = 0.085	*w* = 1/[σ^2^(*F*_o_^2^) + (0.0418*P*)^2^ + 2.0842*P*] where *P* = (*F*_o_^2^ + 2*F*_c_^2^)/3
*S* = 1.10	(Δ/σ)_max_ = 0.001
13317 reflections	Δρ_max_ = 0.64 e Å^−^^3^
839 parameters	Δρ_min_ = −0.58 e Å^−^^3^
0 restraints	Extinction correction: *SHELXL97* (Sheldrick, 2008), Fc^*^=kFc[1+0.001xFc^2^λ^3^/sin(2θ)]^-1/4^
Primary atom site location: structure-invariant direct methods	Extinction coefficient: 0.0006 (2)

### Special details

**Table:**

Geometry. All e.s.d.'s (except the e.s.d. in the dihedral angle between two l.s. planes) are estimated using the full covariance matrix. The cell e.s.d.'s are taken into account individually in the estimation of e.s.d.'s in distances, angles and torsion angles; correlations between e.s.d.'s in cell parameters are only used when they are defined by crystal symmetry. An approximate (isotropic) treatment of cell e.s.d.'s is used for estimating e.s.d.'s involving l.s. planes.
Refinement. Refinement of *F*^2^ against ALL reflections. The weighted *R*-factor *wR* and goodness of fit *S* are based on *F*^2^, conventional *R*-factors *R* are based on *F*, with *F* set to zero for negative *F*^2^. The threshold expression of *F*^2^ > σ(*F*^2^) is used only for calculating *R*-factors(gt) *etc*. and is not relevant to the choice of reflections for refinement. *R*-factors based on *F*^2^ are statistically about twice as large as those based on *F*, and *R*- factors based on ALL data will be even larger.

### Fractional atomic coordinates and isotropic or equivalent isotropic displacement parameters (Å^2^)

**Table:**

	*x*	*y*	*z*	*U*_iso_*/*U*_eq_	
Cd	0.192877 (10)	0.283327 (9)	0.485889 (9)	0.01474 (5)	
O1	0.06337 (11)	0.32544 (9)	0.35219 (9)	0.0228 (3)	
O2	0.29101 (10)	0.27697 (10)	0.64582 (9)	0.0206 (3)	
N1	0.14498 (12)	0.42841 (11)	0.51244 (10)	0.0167 (3)	
N5	0.10987 (12)	0.18992 (11)	0.54874 (11)	0.0172 (3)	
N3	0.17957 (12)	0.19067 (11)	0.35573 (10)	0.0169 (3)	
N6	0.07496 (13)	0.12902 (12)	0.65733 (11)	0.0198 (3)	
N13	0.19867 (13)	0.42562 (14)	0.16615 (12)	0.0269 (4)	
N4	0.12475 (13)	0.13808 (12)	0.20952 (11)	0.0204 (3)	
O14	0.20300 (12)	0.46536 (12)	0.24164 (10)	0.0323 (4)	
N8	0.52146 (12)	0.35124 (12)	0.60364 (11)	0.0196 (3)	
O13	0.21802 (14)	0.34418 (12)	0.15124 (12)	0.0383 (4)	
N2	0.09934 (12)	0.56535 (11)	0.47420 (11)	0.0179 (3)	
C17	0.05792 (16)	0.63423 (14)	0.41886 (14)	0.0212 (4)	
H17A	0.0304	0.6812	0.4529	0.025*	
H17B	0.0019	0.6004	0.3658	0.025*	
C2	0.22514 (15)	0.49398 (14)	0.67745 (13)	0.0208 (4)	
H2A	0.2427	0.4357	0.6948	0.025*	
N7	0.35983 (12)	0.32424 (12)	0.52112 (11)	0.0185 (3)	
C28	0.24788 (15)	0.22019 (14)	0.69402 (13)	0.0205 (4)	
H28A	0.2460	0.2593	0.7493	0.025*	
H28B	0.2872	0.1680	0.7098	0.025*	
C8	0.05114 (15)	0.42257 (13)	0.35234 (13)	0.0183 (4)	
H8A	−0.0211	0.4291	0.3306	0.022*	
H8B	0.0850	0.4500	0.3140	0.022*	
C25	−0.10309 (16)	0.04850 (15)	0.56962 (15)	0.0241 (4)	
H25A	−0.1180	0.0231	0.6162	0.029*	
C36	0.41620 (15)	0.35206 (14)	0.46889 (13)	0.0200 (4)	
C22	−0.05747 (15)	0.12440 (14)	0.42964 (13)	0.0203 (4)	
H22A	−0.0422	0.1492	0.3829	0.024*	
O10	0.06682 (14)	0.61831 (13)	−0.11392 (11)	0.0403 (4)	
C12	0.23575 (18)	0.00999 (16)	0.20566 (15)	0.0279 (5)	
H12A	0.2110	−0.0131	0.1434	0.034*	
C27	0.14365 (15)	0.18099 (13)	0.63318 (12)	0.0174 (4)	
C9	0.04463 (16)	0.26994 (14)	0.26631 (13)	0.0223 (4)	
H9A	0.0557	0.3111	0.2248	0.027*	
H9B	−0.0256	0.2367	0.2423	0.027*	
C21	0.01217 (14)	0.14003 (13)	0.51479 (13)	0.0175 (4)	
C15	0.31005 (15)	0.08006 (15)	0.39287 (14)	0.0226 (4)	
H15A	0.3353	0.1034	0.4551	0.027*	
C10	0.11672 (15)	0.19966 (13)	0.27802 (12)	0.0178 (4)	
C16	0.23325 (15)	0.11831 (13)	0.33660 (13)	0.0181 (4)	
C29	0.39802 (14)	0.29296 (15)	0.67509 (13)	0.0205 (4)	
H29A	0.4253	0.2338	0.6887	0.025*	
H29B	0.4249	0.3432	0.7290	0.025*	
C24	−0.17214 (16)	0.03415 (15)	0.48503 (15)	0.0261 (4)	
H24A	−0.2365	−0.0018	0.4733	0.031*	
C11	0.19855 (15)	0.08412 (14)	0.24534 (13)	0.0206 (4)	
C31	0.51809 (15)	0.36926 (14)	0.52038 (14)	0.0210 (4)	
C1	0.17745 (14)	0.49915 (13)	0.58885 (13)	0.0177 (4)	
C6	0.15063 (14)	0.58593 (14)	0.56497 (13)	0.0186 (4)	
N14	0.08943 (15)	0.71026 (14)	0.06366 (14)	0.0318 (4)	
C7	0.09864 (14)	0.47134 (13)	0.44684 (13)	0.0164 (4)	
O12	0.17701 (18)	0.38220 (15)	−0.15725 (14)	0.0538 (5)	
C23	−0.15003 (16)	0.07122 (14)	0.41583 (14)	0.0238 (4)	
H23A	−0.1995	0.0596	0.3585	0.029*	
O15	0.14505 (16)	0.76137 (14)	0.13161 (14)	0.0516 (5)	
C3	0.24581 (16)	0.57717 (15)	0.73893 (14)	0.0254 (4)	
H3A	0.2772	0.5756	0.8000	0.030*	
C37	0.08652 (17)	0.10619 (16)	0.74567 (14)	0.0254 (4)	
H37A	0.1582	0.1226	0.7832	0.031*	
H37B	0.0664	0.0369	0.7398	0.031*	
C26	−0.01042 (15)	0.10222 (13)	0.58284 (13)	0.0193 (4)	
C39	0.61246 (15)	0.34820 (15)	0.67757 (14)	0.0243 (4)	
H39A	0.5992	0.3643	0.7345	0.029*	
H39B	0.6675	0.3964	0.6775	0.029*	
O16	0.01603 (16)	0.73527 (14)	0.01351 (13)	0.0488 (5)	
C51	0.14575 (15)	0.56758 (15)	0.11058 (14)	0.0235 (4)	
H51A	0.1544	0.5977	0.1695	0.028*	
C35	0.38574 (17)	0.36385 (16)	0.38061 (14)	0.0272 (4)	
H35A	0.3166	0.3530	0.3453	0.033*	
C32	0.59313 (16)	0.39831 (16)	0.48618 (15)	0.0264 (4)	
H32A	0.6623	0.4102	0.5215	0.032*	
C5	0.17423 (16)	0.67040 (14)	0.62663 (14)	0.0237 (4)	
H5A	0.1584	0.7292	0.6094	0.028*	
O11	0.03647 (18)	0.43433 (16)	−0.20943 (13)	0.0574 (6)	
C19	0.06537 (17)	0.13018 (15)	0.11550 (13)	0.0267 (4)	
H19A	0.1076	0.1133	0.0783	0.032*	
H19B	0.0444	0.1926	0.1050	0.032*	
C14	0.34766 (17)	0.00645 (16)	0.35367 (16)	0.0286 (5)	
H14A	0.3999	−0.0213	0.3901	0.034*	
C38	0.0229 (2)	0.16014 (18)	0.79053 (16)	0.0346 (5)	
H38A	0.0323	0.1433	0.8491	0.052*	
H38B	−0.0483	0.1432	0.7540	0.052*	
H38C	0.0434	0.2288	0.7974	0.052*	
C34	0.46017 (18)	0.39198 (18)	0.34663 (16)	0.0324 (5)	
H34A	0.4418	0.4001	0.2865	0.039*	
C47	0.09519 (16)	0.57532 (16)	−0.05170 (14)	0.0273 (5)	
C33	0.56212 (18)	0.40887 (17)	0.39857 (16)	0.0326 (5)	
H33A	0.6111	0.4281	0.3727	0.039*	
C13	0.31112 (18)	−0.02821 (16)	0.26226 (16)	0.0306 (5)	
H13A	0.3386	−0.0793	0.2383	0.037*	
C30	0.42526 (14)	0.32394 (13)	0.59926 (13)	0.0182 (4)	
C18	0.13702 (18)	0.68583 (17)	0.38971 (16)	0.0315 (5)	
H18A	0.1067	0.7306	0.3524	0.047*	
H18B	0.1641	0.6395	0.3557	0.047*	
H18C	0.1916	0.7209	0.4421	0.047*	
C40	0.64495 (17)	0.25090 (17)	0.67047 (16)	0.0304 (5)	
H40A	0.7057	0.2510	0.7207	0.046*	
H40B	0.6593	0.2354	0.6147	0.046*	
H40C	0.5909	0.2032	0.6714	0.046*	
N12	0.11204 (18)	0.42930 (16)	−0.14835 (14)	0.0380 (5)	
C52	0.11053 (16)	0.61404 (15)	0.04106 (14)	0.0248 (4)	
C48	0.12439 (17)	0.47993 (17)	−0.05982 (14)	0.0280 (5)	
C4	0.22181 (16)	0.66428 (15)	0.71403 (14)	0.0260 (4)	
H4A	0.2388	0.7201	0.7584	0.031*	
C50	0.16865 (15)	0.47630 (15)	0.09423 (14)	0.0235 (4)	
C20	−0.02683 (18)	0.0556 (2)	0.08858 (16)	0.0381 (6)	
H20A	−0.0648	0.0519	0.0257	0.057*	
H20B	−0.0694	0.0728	0.1246	0.057*	
H20C	−0.0062	−0.0064	0.0980	0.057*	
O3	0.60335 (14)	0.85519 (12)	0.11334 (12)	0.0396 (4)	
O6	0.40616 (15)	0.46250 (12)	−0.13643 (12)	0.0398 (4)	
O4	0.44373 (16)	0.79884 (15)	0.15928 (14)	0.0510 (5)	
N10	0.47922 (17)	0.51787 (14)	−0.13474 (13)	0.0345 (5)	
N11	0.70229 (15)	0.81938 (16)	−0.01224 (14)	0.0354 (5)	
O9	0.77132 (18)	0.8475 (3)	0.05314 (16)	0.0918 (11)	
C42	0.48590 (16)	0.71005 (15)	0.04803 (13)	0.0231 (4)	
N9	0.42620 (15)	0.72432 (14)	0.10673 (12)	0.0297 (4)	
O5	0.35880 (17)	0.66104 (15)	0.10079 (15)	0.0568 (6)	
C43	0.45710 (17)	0.62643 (15)	−0.01328 (14)	0.0257 (4)	
H43A	0.4016	0.5822	−0.0149	0.031*	
O8	0.69775 (17)	0.84159 (17)	−0.08432 (16)	0.0599 (6)	
C46	0.61808 (17)	0.75269 (16)	−0.01001 (15)	0.0274 (4)	
O7	0.53003 (19)	0.50084 (14)	−0.18415 (14)	0.0545 (6)	
C41	0.57210 (17)	0.78032 (15)	0.05784 (14)	0.0261 (4)	
C44	0.50875 (18)	0.60680 (15)	−0.07235 (14)	0.0267 (4)	
C45	0.58954 (18)	0.67155 (17)	−0.07216 (15)	0.0304 (5)	
H45A	0.6234	0.6591	−0.1143	0.036*	
O18	0.40567 (15)	0.06023 (13)	0.63637 (14)	0.0481 (5)	
N16	0.43351 (16)	−0.07840 (15)	0.68649 (17)	0.0418 (5)	
C56	0.42918 (18)	0.01539 (19)	0.69660 (19)	0.0397 (6)	
H56A	0.4458	0.0497	0.7555	0.048*	
C58	0.4688 (3)	−0.1241 (3)	0.7626 (3)	0.0718 (10)	
H58A	0.4810	−0.0779	0.8173	0.108*	
H58B	0.5316	−0.1482	0.7630	0.108*	
H58C	0.4178	−0.1773	0.7591	0.108*	
C57	0.4112 (2)	−0.1361 (2)	0.5991 (2)	0.0539 (8)	
H57A	0.3876	−0.0973	0.5536	0.081*	
H57B	0.3588	−0.1899	0.5907	0.081*	
H57C	0.4723	−0.1602	0.5941	0.081*	
O17	0.82180 (15)	0.11818 (12)	0.21533 (11)	0.0388 (4)	
N15	0.71324 (15)	0.10569 (14)	0.07483 (14)	0.0331 (4)	
C53	0.74929 (18)	0.07703 (17)	0.15266 (17)	0.0314 (5)	
H53A	0.7155	0.0197	0.1597	0.038*	
C55	0.7593 (2)	0.19250 (19)	0.05772 (18)	0.0405 (6)	
H55A	0.8147	0.2242	0.1111	0.061*	
H55B	0.7089	0.2350	0.0420	0.061*	
H55C	0.7855	0.1769	0.0085	0.061*	
C54	0.6277 (2)	0.0506 (2)	0.0023 (2)	0.0578 (9)	
H54A	0.6024	−0.0058	0.0213	0.087*	
H54B	0.6491	0.0306	−0.0491	0.087*	
H54C	0.5742	0.0899	−0.0141	0.087*	
C49	0.16003 (16)	0.43299 (16)	0.00900 (15)	0.0265 (4)	
H49A	0.1786	0.3716	−0.0015	0.032*	

### Atomic displacement parameters (Å^2^)

**Table:**

	*U*^11^	*U*^22^	*U*^33^	*U*^12^	*U*^13^	*U*^23^
Cd	0.01519 (7)	0.01451 (7)	0.01395 (7)	0.00324 (5)	0.00374 (5)	0.00237 (5)
O1	0.0339 (8)	0.0148 (6)	0.0169 (7)	0.0083 (6)	0.0038 (6)	0.0009 (5)
O2	0.0157 (6)	0.0250 (7)	0.0219 (7)	0.0024 (6)	0.0049 (5)	0.0097 (6)
N1	0.0182 (8)	0.0151 (7)	0.0172 (8)	0.0044 (6)	0.0061 (6)	0.0023 (6)
N5	0.0184 (8)	0.0141 (7)	0.0188 (8)	0.0031 (6)	0.0055 (6)	0.0023 (6)
N3	0.0178 (8)	0.0157 (7)	0.0166 (8)	0.0028 (6)	0.0048 (6)	0.0023 (6)
N6	0.0226 (8)	0.0189 (8)	0.0191 (8)	0.0026 (7)	0.0079 (7)	0.0053 (6)
N13	0.0193 (8)	0.0341 (10)	0.0277 (10)	0.0024 (8)	0.0057 (7)	0.0122 (8)
N4	0.0253 (9)	0.0200 (8)	0.0142 (8)	0.0050 (7)	0.0045 (7)	0.0007 (6)
O14	0.0302 (8)	0.0442 (10)	0.0216 (8)	0.0018 (7)	0.0061 (6)	0.0115 (7)
N8	0.0144 (8)	0.0202 (8)	0.0216 (8)	0.0014 (6)	0.0028 (6)	0.0031 (6)
O13	0.0410 (10)	0.0353 (9)	0.0423 (10)	0.0138 (8)	0.0120 (8)	0.0171 (8)
N2	0.0201 (8)	0.0151 (7)	0.0196 (8)	0.0050 (6)	0.0071 (7)	0.0038 (6)
C17	0.0250 (10)	0.0169 (9)	0.0243 (10)	0.0084 (8)	0.0088 (8)	0.0068 (8)
C2	0.0236 (10)	0.0198 (9)	0.0196 (10)	0.0057 (8)	0.0074 (8)	0.0038 (8)
N7	0.0160 (8)	0.0192 (8)	0.0193 (8)	0.0013 (6)	0.0047 (6)	0.0038 (6)
C28	0.0213 (10)	0.0233 (10)	0.0172 (9)	0.0019 (8)	0.0063 (8)	0.0054 (8)
C8	0.0227 (9)	0.0154 (9)	0.0184 (9)	0.0061 (8)	0.0075 (8)	0.0042 (7)
C25	0.0248 (10)	0.0198 (10)	0.0311 (11)	0.0028 (8)	0.0124 (9)	0.0080 (8)
C36	0.0179 (9)	0.0196 (9)	0.0216 (10)	0.0024 (8)	0.0053 (8)	0.0033 (7)
C22	0.0213 (10)	0.0169 (9)	0.0224 (10)	0.0040 (8)	0.0060 (8)	0.0045 (7)
O10	0.0497 (11)	0.0429 (10)	0.0244 (8)	−0.0011 (9)	0.0046 (8)	0.0163 (8)
C12	0.0343 (12)	0.0252 (10)	0.0247 (11)	0.0052 (9)	0.0124 (9)	−0.0008 (8)
C27	0.0216 (9)	0.0147 (8)	0.0178 (9)	0.0057 (7)	0.0083 (7)	0.0031 (7)
C9	0.0275 (10)	0.0183 (9)	0.0164 (9)	0.0056 (8)	0.0013 (8)	−0.0002 (7)
C21	0.0182 (9)	0.0132 (8)	0.0218 (10)	0.0034 (7)	0.0075 (8)	0.0027 (7)
C15	0.0218 (10)	0.0230 (10)	0.0221 (10)	0.0033 (8)	0.0057 (8)	0.0048 (8)
C10	0.0201 (9)	0.0148 (8)	0.0166 (9)	0.0000 (7)	0.0046 (7)	0.0011 (7)
C16	0.0201 (9)	0.0155 (9)	0.0202 (9)	0.0018 (7)	0.0092 (8)	0.0025 (7)
C29	0.0168 (9)	0.0231 (10)	0.0181 (9)	0.0010 (8)	0.0022 (7)	0.0020 (7)
C24	0.0212 (10)	0.0190 (10)	0.0369 (12)	−0.0001 (8)	0.0091 (9)	0.0047 (9)
C11	0.0225 (10)	0.0177 (9)	0.0222 (10)	0.0024 (8)	0.0081 (8)	0.0043 (7)
C31	0.0199 (9)	0.0184 (9)	0.0235 (10)	0.0026 (8)	0.0060 (8)	0.0030 (8)
C1	0.0169 (9)	0.0159 (9)	0.0214 (9)	0.0025 (7)	0.0087 (7)	0.0015 (7)
C6	0.0184 (9)	0.0181 (9)	0.0200 (9)	0.0037 (7)	0.0075 (7)	0.0030 (7)
N14	0.0329 (10)	0.0322 (10)	0.0355 (11)	0.0080 (9)	0.0143 (9)	0.0134 (9)
C7	0.0176 (9)	0.0143 (8)	0.0201 (9)	0.0041 (7)	0.0091 (7)	0.0045 (7)
O12	0.0704 (14)	0.0518 (12)	0.0456 (12)	0.0072 (11)	0.0337 (11)	−0.0018 (9)
C23	0.0214 (10)	0.0198 (9)	0.0262 (11)	0.0030 (8)	0.0033 (8)	0.0015 (8)
O15	0.0498 (12)	0.0406 (11)	0.0516 (12)	0.0127 (9)	0.0050 (10)	−0.0106 (9)
C3	0.0267 (11)	0.0275 (11)	0.0190 (10)	0.0052 (9)	0.0049 (8)	−0.0001 (8)
C37	0.0307 (11)	0.0274 (11)	0.0220 (10)	0.0056 (9)	0.0109 (9)	0.0109 (8)
C26	0.0220 (10)	0.0159 (9)	0.0213 (10)	0.0054 (8)	0.0083 (8)	0.0033 (7)
C39	0.0164 (9)	0.0292 (11)	0.0215 (10)	0.0009 (8)	0.0002 (8)	0.0020 (8)
O16	0.0551 (12)	0.0477 (11)	0.0463 (11)	0.0221 (10)	0.0096 (9)	0.0228 (9)
C51	0.0195 (9)	0.0297 (11)	0.0212 (10)	−0.0003 (8)	0.0074 (8)	0.0053 (8)
C35	0.0248 (10)	0.0314 (11)	0.0240 (11)	0.0011 (9)	0.0052 (9)	0.0085 (9)
C32	0.0195 (10)	0.0272 (11)	0.0321 (12)	0.0003 (8)	0.0091 (9)	0.0047 (9)
C5	0.0264 (10)	0.0158 (9)	0.0285 (11)	0.0059 (8)	0.0090 (9)	0.0012 (8)
O11	0.0684 (14)	0.0672 (14)	0.0218 (9)	−0.0017 (11)	0.0013 (9)	−0.0008 (9)
C19	0.0364 (12)	0.0263 (10)	0.0135 (9)	0.0062 (9)	0.0036 (8)	0.0006 (8)
C14	0.0249 (11)	0.0271 (11)	0.0362 (12)	0.0101 (9)	0.0098 (9)	0.0096 (9)
C38	0.0453 (14)	0.0373 (13)	0.0268 (12)	0.0089 (11)	0.0185 (11)	0.0070 (10)
C34	0.0349 (12)	0.0376 (13)	0.0255 (11)	−0.0004 (10)	0.0111 (10)	0.0094 (10)
C47	0.0237 (10)	0.0319 (11)	0.0236 (11)	−0.0059 (9)	0.0047 (8)	0.0097 (9)
C33	0.0321 (12)	0.0341 (12)	0.0370 (13)	0.0015 (10)	0.0188 (10)	0.0089 (10)
C13	0.0361 (12)	0.0238 (11)	0.0368 (13)	0.0126 (10)	0.0174 (10)	0.0037 (9)
C30	0.0167 (9)	0.0151 (8)	0.0201 (9)	0.0016 (7)	0.0037 (7)	0.0006 (7)
C18	0.0340 (12)	0.0288 (11)	0.0354 (13)	0.0050 (10)	0.0121 (10)	0.0153 (10)
C40	0.0214 (10)	0.0341 (12)	0.0376 (13)	0.0086 (9)	0.0081 (9)	0.0136 (10)
N12	0.0496 (13)	0.0375 (11)	0.0265 (11)	−0.0058 (10)	0.0176 (10)	0.0009 (9)
C52	0.0222 (10)	0.0259 (10)	0.0262 (11)	0.0014 (8)	0.0070 (8)	0.0076 (8)
C48	0.0286 (11)	0.0333 (12)	0.0201 (10)	−0.0043 (9)	0.0087 (9)	0.0024 (9)
C4	0.0285 (11)	0.0222 (10)	0.0229 (10)	0.0030 (9)	0.0067 (9)	−0.0052 (8)
C50	0.0202 (10)	0.0294 (11)	0.0210 (10)	0.0011 (8)	0.0062 (8)	0.0085 (8)
C20	0.0301 (12)	0.0532 (16)	0.0231 (11)	0.0005 (11)	0.0059 (10)	−0.0081 (10)
O3	0.0487 (11)	0.0304 (9)	0.0374 (10)	−0.0051 (8)	0.0196 (8)	−0.0057 (7)
O6	0.0499 (11)	0.0253 (8)	0.0350 (10)	0.0002 (8)	0.0027 (8)	0.0043 (7)
O4	0.0541 (12)	0.0509 (12)	0.0484 (12)	−0.0051 (10)	0.0319 (10)	−0.0149 (9)
N10	0.0520 (13)	0.0264 (10)	0.0229 (10)	0.0136 (10)	0.0067 (9)	0.0047 (8)
N11	0.0281 (10)	0.0405 (11)	0.0386 (12)	0.0029 (9)	0.0143 (9)	0.0040 (9)
O9	0.0456 (13)	0.158 (3)	0.0460 (14)	−0.0456 (16)	−0.0012 (11)	0.0117 (16)
C42	0.0266 (10)	0.0261 (10)	0.0173 (9)	0.0074 (9)	0.0064 (8)	0.0064 (8)
N9	0.0326 (10)	0.0351 (10)	0.0233 (9)	0.0070 (9)	0.0098 (8)	0.0082 (8)
O5	0.0661 (14)	0.0498 (12)	0.0623 (14)	−0.0150 (11)	0.0440 (12)	−0.0002 (10)
C43	0.0300 (11)	0.0243 (10)	0.0201 (10)	0.0037 (9)	0.0029 (8)	0.0075 (8)
O8	0.0517 (13)	0.0697 (15)	0.0641 (15)	−0.0002 (11)	0.0209 (11)	0.0323 (12)
C46	0.0245 (11)	0.0307 (11)	0.0278 (11)	0.0052 (9)	0.0081 (9)	0.0078 (9)
O7	0.0890 (17)	0.0351 (10)	0.0461 (12)	0.0100 (11)	0.0369 (12)	−0.0044 (9)
C41	0.0306 (11)	0.0250 (10)	0.0223 (10)	0.0059 (9)	0.0073 (9)	0.0052 (8)
C44	0.0354 (12)	0.0224 (10)	0.0187 (10)	0.0081 (9)	0.0034 (9)	0.0026 (8)
C45	0.0340 (12)	0.0360 (12)	0.0245 (11)	0.0139 (10)	0.0113 (9)	0.0069 (9)
O18	0.0396 (10)	0.0337 (10)	0.0564 (13)	0.0088 (8)	−0.0009 (9)	−0.0034 (9)
N16	0.0280 (10)	0.0329 (11)	0.0558 (14)	0.0032 (9)	0.0062 (10)	−0.0008 (10)
C56	0.0246 (11)	0.0360 (13)	0.0470 (15)	0.0004 (10)	0.0052 (11)	−0.0115 (12)
C58	0.068 (2)	0.059 (2)	0.078 (3)	0.0037 (18)	0.007 (2)	0.0236 (19)
C57	0.0418 (15)	0.0357 (14)	0.070 (2)	0.0040 (12)	0.0109 (15)	−0.0168 (14)
O17	0.0541 (11)	0.0317 (9)	0.0279 (9)	0.0028 (8)	0.0117 (8)	0.0031 (7)
N15	0.0271 (10)	0.0269 (10)	0.0425 (12)	−0.0008 (8)	0.0066 (9)	0.0109 (9)
C53	0.0333 (12)	0.0267 (11)	0.0409 (14)	0.0079 (10)	0.0189 (11)	0.0101 (10)
C55	0.0412 (14)	0.0365 (13)	0.0407 (14)	−0.0018 (11)	0.0071 (11)	0.0166 (11)
C54	0.0373 (15)	0.0492 (17)	0.069 (2)	−0.0086 (13)	−0.0079 (14)	0.0203 (16)
C49	0.0221 (10)	0.0286 (11)	0.0288 (11)	0.0004 (9)	0.0096 (9)	0.0041 (9)

### Geometric parameters (Å, °)

**Table:**

Cd—N5	2.2387 (16)	C37—H37B	0.9900
Cd—N7	2.2439 (16)	C39—C40	1.510 (3)
Cd—N3	2.2578 (16)	C39—H39A	0.9900
Cd—N1	2.2725 (15)	C39—H39B	0.9900
Cd—O2	2.5390 (14)	C51—C52	1.371 (3)
Cd—O1	2.5494 (14)	C51—C50	1.387 (3)
O1—C8	1.420 (2)	C51—H51A	0.9500
O1—C9	1.425 (2)	C35—C34	1.379 (3)
O2—C28	1.419 (2)	C35—H35A	0.9500
O2—C29	1.421 (2)	C32—C33	1.377 (3)
N1—C7	1.327 (2)	C32—H32A	0.9500
N1—C1	1.398 (2)	C5—C4	1.379 (3)
N5—C27	1.324 (2)	C5—H5A	0.9500
N5—C21	1.395 (2)	O11—N12	1.224 (3)
N3—C10	1.324 (2)	C19—C20	1.509 (3)
N3—C16	1.396 (2)	C19—H19A	0.9900
N6—C27	1.351 (3)	C19—H19B	0.9900
N6—C26	1.387 (3)	C14—C13	1.396 (3)
N6—C37	1.469 (2)	C14—H14A	0.9500
N13—O13	1.232 (3)	C38—H38A	0.9800
N13—O14	1.246 (2)	C38—H38B	0.9800
N13—C50	1.431 (3)	C38—H38C	0.9800
N4—C10	1.351 (2)	C34—C33	1.400 (3)
N4—C11	1.389 (3)	C34—H34A	0.9500
N4—C19	1.468 (3)	C47—C52	1.457 (3)
N8—C30	1.354 (2)	C47—C48	1.466 (3)
N8—C31	1.389 (3)	C33—H33A	0.9500
N8—C39	1.467 (2)	C13—H13A	0.9500
N2—C7	1.355 (2)	C18—H18A	0.9800
N2—C6	1.389 (3)	C18—H18B	0.9800
N2—C17	1.468 (2)	C18—H18C	0.9800
C17—C18	1.510 (3)	C40—H40A	0.9800
C17—H17A	0.9900	C40—H40B	0.9800
C17—H17B	0.9900	C40—H40C	0.9800
C2—C3	1.379 (3)	N12—C48	1.454 (3)
C2—C1	1.393 (3)	C48—C49	1.368 (3)
C2—H2A	0.9500	C4—H4A	0.9500
N7—C30	1.312 (2)	C50—C49	1.385 (3)
N7—C36	1.396 (2)	C20—H20A	0.9800
C28—C27	1.494 (3)	C20—H20B	0.9800
C28—H28A	0.9900	C20—H20C	0.9800
C28—H28B	0.9900	O3—C41	1.239 (3)
C8—C7	1.492 (3)	O6—N10	1.230 (3)
C8—H8A	0.9900	O4—N9	1.216 (3)
C8—H8B	0.9900	N10—O7	1.240 (3)
C25—C24	1.381 (3)	N10—C44	1.442 (3)
C25—C26	1.392 (3)	N11—O9	1.179 (3)
C25—H25A	0.9500	N11—O8	1.232 (3)
C36—C35	1.392 (3)	N11—C46	1.465 (3)
C36—C31	1.398 (3)	C42—C43	1.377 (3)
C22—C23	1.385 (3)	C42—N9	1.454 (3)
C22—C21	1.390 (3)	C42—C41	1.456 (3)
C22—H22A	0.9500	N9—O5	1.221 (3)
O10—C47	1.234 (3)	C43—C44	1.380 (3)
C12—C13	1.386 (3)	C43—H43A	0.9500
C12—C11	1.390 (3)	C46—C45	1.358 (3)
C12—H12A	0.9500	C46—C41	1.461 (3)
C9—C10	1.491 (3)	C44—C45	1.399 (3)
C9—H9A	0.9900	C45—H45A	0.9500
C9—H9B	0.9900	O18—C56	1.219 (3)
C21—C26	1.396 (3)	N16—C56	1.341 (3)
C15—C14	1.385 (3)	N16—C58	1.449 (4)
C15—C16	1.396 (3)	N16—C57	1.450 (4)
C15—H15A	0.9500	C56—H56A	0.9500
C16—C11	1.393 (3)	C58—H58A	0.9800
C29—C30	1.499 (3)	C58—H58B	0.9800
C29—H29A	0.9900	C58—H58C	0.9800
C29—H29B	0.9900	C57—H57A	0.9800
C24—C23	1.405 (3)	C57—H57B	0.9800
C24—H24A	0.9500	C57—H57C	0.9800
C31—C32	1.393 (3)	O17—C53	1.220 (3)
C1—C6	1.407 (3)	N15—C53	1.339 (3)
C6—C5	1.391 (3)	N15—C55	1.450 (3)
N14—O15	1.216 (3)	N15—C54	1.458 (3)
N14—O16	1.222 (3)	C53—H53A	0.9500
N14—C52	1.458 (3)	C55—H55A	0.9800
O12—N12	1.221 (3)	C55—H55B	0.9800
C23—H23A	0.9500	C55—H55C	0.9800
C3—C4	1.407 (3)	C54—H54A	0.9800
C3—H3A	0.9500	C54—H54B	0.9800
C37—C38	1.517 (3)	C54—H54C	0.9800
C37—H37A	0.9900	C49—H49A	0.9500
			
N5—Cd—N7	128.27 (6)	C40—C39—H39A	109.4
N5—Cd—N3	103.76 (6)	N8—C39—H39B	109.4
N7—Cd—N3	92.90 (6)	C40—C39—H39B	109.4
N5—Cd—N1	104.47 (6)	H39A—C39—H39B	108.0
N7—Cd—N1	100.89 (6)	C52—C51—C50	119.5 (2)
N3—Cd—N1	129.81 (6)	C52—C51—H51A	120.2
N5—Cd—O2	66.35 (5)	C50—C51—H51A	120.2
N7—Cd—O2	66.30 (5)	C34—C35—C36	117.2 (2)
N3—Cd—O2	132.15 (5)	C34—C35—H35A	121.4
N1—Cd—O2	97.19 (5)	C36—C35—H35A	121.4
N5—Cd—O1	107.57 (5)	C33—C32—C31	116.7 (2)
N7—Cd—O1	123.92 (5)	C33—C32—H32A	121.7
N3—Cd—O1	66.55 (5)	C31—C32—H32A	121.7
N1—Cd—O1	65.74 (5)	C4—C5—C6	116.45 (19)
O2—Cd—O1	160.61 (5)	C4—C5—H5A	121.8
C8—O1—C9	114.53 (14)	C6—C5—H5A	121.8
C8—O1—Cd	118.48 (11)	N4—C19—C20	111.37 (18)
C9—O1—Cd	119.17 (11)	N4—C19—H19A	109.4
C28—O2—C29	115.00 (14)	C20—C19—H19A	109.4
C28—O2—Cd	119.89 (11)	N4—C19—H19B	109.4
C29—O2—Cd	119.70 (11)	C20—C19—H19B	109.4
C7—N1—C1	105.45 (15)	H19A—C19—H19B	108.0
C7—N1—Cd	121.79 (12)	C15—C14—C13	122.0 (2)
C1—N1—Cd	130.56 (13)	C15—C14—H14A	119.0
C27—N5—C21	105.84 (16)	C13—C14—H14A	119.0
C27—N5—Cd	124.02 (13)	C37—C38—H38A	109.5
C21—N5—Cd	129.72 (12)	C37—C38—H38B	109.5
C10—N3—C16	105.45 (16)	H38A—C38—H38B	109.5
C10—N3—Cd	123.70 (13)	C37—C38—H38C	109.5
C16—N3—Cd	130.80 (13)	H38A—C38—H38C	109.5
C27—N6—C26	106.92 (16)	H38B—C38—H38C	109.5
C27—N6—C37	127.10 (18)	C35—C34—C33	121.7 (2)
C26—N6—C37	125.97 (17)	C35—C34—H34A	119.2
O13—N13—O14	122.40 (18)	C33—C34—H34A	119.2
O13—N13—C50	119.10 (19)	O10—C47—C52	125.0 (2)
O14—N13—C50	118.50 (19)	O10—C47—C48	124.7 (2)
C10—N4—C11	106.97 (16)	C52—C47—C48	110.15 (18)
C10—N4—C19	125.83 (17)	C32—C33—C34	121.7 (2)
C11—N4—C19	127.20 (17)	C32—C33—H33A	119.2
C30—N8—C31	106.87 (16)	C34—C33—H33A	119.2
C30—N8—C39	126.30 (17)	C12—C13—C14	121.7 (2)
C31—N8—C39	126.23 (17)	C12—C13—H13A	119.2
C7—N2—C6	106.96 (15)	C14—C13—H13A	119.2
C7—N2—C17	127.17 (17)	N7—C30—N8	112.83 (17)
C6—N2—C17	125.82 (16)	N7—C30—C29	124.15 (17)
N2—C17—C18	111.60 (17)	N8—C30—C29	122.97 (17)
N2—C17—H17A	109.3	C17—C18—H18A	109.5
C18—C17—H17A	109.3	C17—C18—H18B	109.5
N2—C17—H17B	109.3	H18A—C18—H18B	109.5
C18—C17—H17B	109.3	C17—C18—H18C	109.5
H17A—C17—H17B	108.0	H18A—C18—H18C	109.5
C3—C2—C1	117.11 (18)	H18B—C18—H18C	109.5
C3—C2—H2A	121.4	C39—C40—H40A	109.5
C1—C2—H2A	121.4	C39—C40—H40B	109.5
C30—N7—C36	105.90 (16)	H40A—C40—H40B	109.5
C30—N7—Cd	124.20 (13)	C39—C40—H40C	109.5
C36—N7—Cd	129.89 (13)	H40A—C40—H40C	109.5
O2—C28—C27	105.36 (15)	H40B—C40—H40C	109.5
O2—C28—H28A	110.7	O12—N12—O11	123.5 (2)
C27—C28—H28A	110.7	O12—N12—C48	117.9 (2)
O2—C28—H28B	110.7	O11—N12—C48	118.6 (2)
C27—C28—H28B	110.7	C51—C52—C47	125.0 (2)
H28A—C28—H28B	108.8	C51—C52—N14	116.3 (2)
O1—C8—C7	105.29 (15)	C47—C52—N14	118.66 (19)
O1—C8—H8A	110.7	C49—C48—N12	116.8 (2)
C7—C8—H8A	110.7	C49—C48—C47	125.4 (2)
O1—C8—H8B	110.7	N12—C48—C47	117.8 (2)
C7—C8—H8B	110.7	C5—C4—C3	121.49 (19)
H8A—C8—H8B	108.8	C5—C4—H4A	119.3
C24—C25—C26	116.38 (19)	C3—C4—H4A	119.3
C24—C25—H25A	121.8	C49—C50—C51	120.80 (19)
C26—C25—H25A	121.8	C49—C50—N13	119.9 (2)
C35—C36—N7	130.54 (19)	C51—C50—N13	119.26 (19)
C35—C36—C31	120.80 (19)	C19—C20—H20A	109.5
N7—C36—C31	108.65 (17)	C19—C20—H20B	109.5
C23—C22—C21	117.25 (18)	H20A—C20—H20B	109.5
C23—C22—H22A	121.4	C19—C20—H20C	109.5
C21—C22—H22A	121.4	H20A—C20—H20C	109.5
C13—C12—C11	116.1 (2)	H20B—C20—H20C	109.5
C13—C12—H12A	122.0	O6—N10—O7	123.1 (2)
C11—C12—H12A	122.0	O6—N10—C44	119.3 (2)
N5—C27—N6	112.57 (17)	O7—N10—C44	117.6 (2)
N5—C27—C28	123.78 (17)	O9—N11—O8	123.2 (2)
N6—C27—C28	123.60 (17)	O9—N11—C46	119.6 (2)
O1—C9—C10	106.28 (16)	O8—N11—C46	117.3 (2)
O1—C9—H9A	110.5	C43—C42—N9	115.8 (2)
C10—C9—H9A	110.5	C43—C42—C41	123.9 (2)
O1—C9—H9B	110.5	N9—C42—C41	120.29 (19)
C10—C9—H9B	110.5	O4—N9—O5	122.0 (2)
H9A—C9—H9B	108.7	O4—N9—C42	119.4 (2)
C22—C21—N5	130.52 (17)	O5—N9—C42	118.6 (2)
C22—C21—C26	120.97 (18)	C42—C43—C44	120.0 (2)
N5—C21—C26	108.51 (17)	C42—C43—H43A	120.0
C14—C15—C16	116.83 (19)	C44—C43—H43A	120.0
C14—C15—H15A	121.6	C45—C46—C41	126.3 (2)
C16—C15—H15A	121.6	C45—C46—N11	116.6 (2)
N3—C10—N4	112.74 (17)	C41—C46—N11	117.1 (2)
N3—C10—C9	124.17 (17)	O3—C41—C42	126.8 (2)
N4—C10—C9	123.09 (17)	O3—C41—C46	122.6 (2)
C11—C16—C15	120.64 (18)	C42—C41—C46	110.58 (19)
C11—C16—N3	108.97 (17)	C43—C44—C45	121.0 (2)
C15—C16—N3	130.39 (18)	C43—C44—N10	119.4 (2)
O2—C29—C30	105.17 (15)	C45—C44—N10	119.6 (2)
O2—C29—H29A	110.7	C46—C45—C44	118.0 (2)
C30—C29—H29A	110.7	C46—C45—H45A	121.0
O2—C29—H29B	110.7	C44—C45—H45A	121.0
C30—C29—H29B	110.7	C56—N16—C58	121.2 (3)
H29A—C29—H29B	108.8	C56—N16—C57	121.2 (3)
C25—C24—C23	121.92 (19)	C58—N16—C57	117.4 (3)
C25—C24—H24A	119.0	O18—C56—N16	125.3 (3)
C23—C24—H24A	119.0	O18—C56—H56A	117.3
N4—C11—C12	131.37 (19)	N16—C56—H56A	117.3
N4—C11—C16	105.86 (17)	N16—C58—H58A	109.5
C12—C11—C16	122.77 (19)	N16—C58—H58B	109.5
N8—C31—C32	132.30 (19)	H58A—C58—H58B	109.5
N8—C31—C36	105.73 (17)	N16—C58—H58C	109.5
C32—C31—C36	121.97 (19)	H58A—C58—H58C	109.5
C2—C1—N1	130.79 (18)	H58B—C58—H58C	109.5
C2—C1—C6	120.42 (18)	N16—C57—H57A	109.5
N1—C1—C6	108.75 (17)	N16—C57—H57B	109.5
N2—C6—C5	131.73 (18)	H57A—C57—H57B	109.5
N2—C6—C1	105.84 (16)	N16—C57—H57C	109.5
C5—C6—C1	122.42 (18)	H57A—C57—H57C	109.5
O15—N14—O16	123.2 (2)	H57B—C57—H57C	109.5
O15—N14—C52	118.6 (2)	C53—N15—C55	121.4 (2)
O16—N14—C52	118.2 (2)	C53—N15—C54	122.1 (2)
N1—C7—N2	112.96 (17)	C55—N15—C54	116.5 (2)
N1—C7—C8	124.20 (17)	O17—C53—N15	125.7 (2)
N2—C7—C8	122.83 (16)	O17—C53—H53A	117.2
C22—C23—C24	121.2 (2)	N15—C53—H53A	117.2
C22—C23—H23A	119.4	N15—C55—H55A	109.5
C24—C23—H23A	119.4	N15—C55—H55B	109.5
C2—C3—C4	122.0 (2)	H55A—C55—H55B	109.5
C2—C3—H3A	119.0	N15—C55—H55C	109.5
C4—C3—H3A	119.0	H55A—C55—H55C	109.5
N6—C37—C38	111.58 (17)	H55B—C55—H55C	109.5
N6—C37—H37A	109.3	N15—C54—H54A	109.5
C38—C37—H37A	109.3	N15—C54—H54B	109.5
N6—C37—H37B	109.3	H54A—C54—H54B	109.5
C38—C37—H37B	109.3	N15—C54—H54C	109.5
H37A—C37—H37B	108.0	H54A—C54—H54C	109.5
N6—C26—C25	131.61 (18)	H54B—C54—H54C	109.5
N6—C26—C21	106.16 (17)	C48—C49—C50	119.0 (2)
C25—C26—C21	122.23 (19)	C48—C49—H49A	120.5
N8—C39—C40	111.29 (17)	C50—C49—H49A	120.5
N8—C39—H39A	109.4		
			
N5—Cd—O1—C8	117.19 (13)	C3—C2—C1—C6	−1.1 (3)
N7—Cd—O1—C8	−68.08 (14)	C7—N1—C1—C2	176.0 (2)
N3—Cd—O1—C8	−145.07 (14)	Cd—N1—C1—C2	−21.1 (3)
N1—Cd—O1—C8	18.86 (12)	C7—N1—C1—C6	−1.6 (2)
O2—Cd—O1—C8	48.8 (2)	Cd—N1—C1—C6	161.36 (13)
N5—Cd—O1—C9	−95.28 (14)	C7—N2—C6—C5	179.5 (2)
N7—Cd—O1—C9	79.45 (15)	C17—N2—C6—C5	1.8 (3)
N3—Cd—O1—C9	2.46 (13)	C7—N2—C6—C1	−1.2 (2)
N1—Cd—O1—C9	166.39 (15)	C17—N2—C6—C1	−178.94 (17)
O2—Cd—O1—C9	−163.70 (14)	C2—C1—C6—N2	−176.11 (17)
N5—Cd—O2—C28	0.26 (13)	N1—C1—C6—N2	1.7 (2)
N7—Cd—O2—C28	−158.27 (15)	C2—C1—C6—C5	3.2 (3)
N3—Cd—O2—C28	−87.10 (14)	N1—C1—C6—C5	−178.95 (18)
N1—Cd—O2—C28	102.95 (13)	C1—N1—C7—N2	0.8 (2)
O1—Cd—O2—C28	75.68 (19)	Cd—N1—C7—N2	−163.99 (12)
N5—Cd—O2—C29	153.02 (14)	C1—N1—C7—C8	−179.72 (17)
N7—Cd—O2—C29	−5.51 (13)	Cd—N1—C7—C8	15.5 (2)
N3—Cd—O2—C29	65.66 (15)	C6—N2—C7—N1	0.3 (2)
N1—Cd—O2—C29	−104.29 (13)	C17—N2—C7—N1	177.95 (17)
O1—Cd—O2—C29	−131.56 (15)	C6—N2—C7—C8	−179.20 (17)
N5—Cd—N1—C7	−119.98 (14)	C17—N2—C7—C8	−1.5 (3)
N7—Cd—N1—C7	105.51 (15)	O1—C8—C7—N1	2.6 (2)
N3—Cd—N1—C7	2.36 (18)	O1—C8—C7—N2	−177.99 (16)
O2—Cd—N1—C7	172.66 (14)	C21—C22—C23—C24	−0.4 (3)
O1—Cd—N1—C7	−16.94 (13)	C25—C24—C23—C22	−0.2 (3)
N5—Cd—N1—C1	79.47 (16)	C1—C2—C3—C4	−1.1 (3)
N7—Cd—N1—C1	−55.04 (16)	C27—N6—C37—C38	108.7 (2)
N3—Cd—N1—C1	−158.19 (14)	C26—N6—C37—C38	−70.0 (3)
O2—Cd—N1—C1	12.11 (16)	C27—N6—C26—C25	179.9 (2)
O1—Cd—N1—C1	−177.49 (17)	C37—N6—C26—C25	−1.2 (3)
N7—Cd—N5—C27	29.95 (18)	C27—N6—C26—C21	0.5 (2)
N3—Cd—N5—C27	134.99 (15)	C37—N6—C26—C21	179.45 (18)
N1—Cd—N5—C27	−86.93 (15)	C24—C25—C26—N6	−179.1 (2)
O2—Cd—N5—C27	4.68 (14)	C24—C25—C26—C21	0.1 (3)
O1—Cd—N5—C27	−155.63 (14)	C22—C21—C26—N6	178.65 (17)
N7—Cd—N5—C21	−158.51 (14)	N5—C21—C26—N6	−0.7 (2)
N3—Cd—N5—C21	−53.46 (16)	C22—C21—C26—C25	−0.8 (3)
N1—Cd—N5—C21	84.61 (16)	N5—C21—C26—C25	179.90 (18)
O2—Cd—N5—C21	176.22 (17)	C30—N8—C39—C40	84.5 (2)
O1—Cd—N5—C21	15.92 (17)	C31—N8—C39—C40	−85.4 (2)
N5—Cd—N3—C10	103.11 (15)	N7—C36—C35—C34	−179.7 (2)
N7—Cd—N3—C10	−126.28 (15)	C31—C36—C35—C34	0.7 (3)
N1—Cd—N3—C10	−19.51 (18)	N8—C31—C32—C33	179.6 (2)
O2—Cd—N3—C10	173.51 (13)	C36—C31—C32—C33	−0.3 (3)
O1—Cd—N3—C10	−0.34 (14)	N2—C6—C5—C4	176.3 (2)
N5—Cd—N3—C16	−79.94 (16)	C1—C6—C5—C4	−2.8 (3)
N7—Cd—N3—C16	50.67 (16)	C10—N4—C19—C20	−93.3 (2)
N1—Cd—N3—C16	157.44 (14)	C11—N4—C19—C20	86.1 (3)
O2—Cd—N3—C16	−9.54 (19)	C16—C15—C14—C13	0.1 (3)
O1—Cd—N3—C16	176.61 (17)	C36—C35—C34—C33	−0.5 (4)
C7—N2—C17—C18	−93.6 (2)	C31—C32—C33—C34	0.5 (3)
C6—N2—C17—C18	83.6 (2)	C35—C34—C33—C32	0.0 (4)
N5—Cd—N7—C30	−23.64 (19)	C11—C12—C13—C14	−0.4 (3)
N3—Cd—N7—C30	−133.73 (16)	C15—C14—C13—C12	0.7 (4)
N1—Cd—N7—C30	94.77 (16)	C36—N7—C30—N8	1.1 (2)
O2—Cd—N7—C30	1.63 (14)	Cd—N7—C30—N8	179.81 (12)
O1—Cd—N7—C30	162.76 (14)	C36—N7—C30—C29	−176.48 (18)
N5—Cd—N7—C36	154.73 (15)	Cd—N7—C30—C29	2.2 (3)
N3—Cd—N7—C36	44.64 (17)	C31—N8—C30—N7	−1.1 (2)
N1—Cd—N7—C36	−86.86 (17)	C39—N8—C30—N7	−172.61 (18)
O2—Cd—N7—C36	180.00 (18)	C31—N8—C30—C29	176.53 (18)
O1—Cd—N7—C36	−18.87 (19)	C39—N8—C30—C29	5.0 (3)
C29—O2—C28—C27	−157.96 (16)	O2—C29—C30—N7	−6.5 (3)
Cd—O2—C28—C27	−3.98 (19)	O2—C29—C30—N8	176.13 (17)
C9—O1—C8—C7	−166.01 (16)	C50—C51—C52—C47	−0.6 (3)
Cd—O1—C8—C7	−17.03 (18)	C50—C51—C52—N14	−179.18 (18)
C30—N7—C36—C35	179.6 (2)	O10—C47—C52—C51	−177.3 (2)
Cd—N7—C36—C35	1.0 (3)	C48—C47—C52—C51	−0.9 (3)
C30—N7—C36—C31	−0.7 (2)	O10—C47—C52—N14	1.2 (3)
Cd—N7—C36—C31	−179.29 (13)	C48—C47—C52—N14	177.70 (18)
C21—N5—C27—N6	−0.2 (2)	O15—N14—C52—C51	37.3 (3)
Cd—N5—C27—N6	173.02 (12)	O16—N14—C52—C51	−142.1 (2)
C21—N5—C27—C28	177.12 (17)	O15—N14—C52—C47	−141.4 (2)
Cd—N5—C27—C28	−9.6 (3)	O16—N14—C52—C47	39.2 (3)
C26—N6—C27—N5	−0.2 (2)	O12—N12—C48—C49	−40.4 (3)
C37—N6—C27—N5	−179.10 (18)	O11—N12—C48—C49	138.1 (2)
C26—N6—C27—C28	−177.54 (18)	O12—N12—C48—C47	141.3 (2)
C37—N6—C27—C28	3.6 (3)	O11—N12—C48—C47	−40.2 (3)
O2—C28—C27—N5	8.4 (3)	O10—C47—C48—C49	177.0 (2)
O2—C28—C27—N6	−174.57 (17)	C52—C47—C48—C49	0.6 (3)
C8—O1—C9—C10	145.05 (16)	O10—C47—C48—N12	−4.8 (3)
Cd—O1—C9—C10	−3.7 (2)	C52—C47—C48—N12	178.71 (19)
C23—C22—C21—N5	−179.95 (19)	C6—C5—C4—C3	0.6 (3)
C23—C22—C21—C26	0.9 (3)	C2—C3—C4—C5	1.4 (3)
C27—N5—C21—C22	−178.7 (2)	C52—C51—C50—C49	2.5 (3)
Cd—N5—C21—C22	8.6 (3)	C52—C51—C50—N13	−175.68 (19)
C27—N5—C21—C26	0.6 (2)	O13—N13—C50—C49	1.1 (3)
Cd—N5—C21—C26	−172.16 (12)	O14—N13—C50—C49	−177.98 (19)
C16—N3—C10—N4	0.5 (2)	O13—N13—C50—C51	179.30 (19)
Cd—N3—C10—N4	178.08 (12)	O14—N13—C50—C51	0.2 (3)
C16—N3—C10—C9	−179.41 (18)	C43—C42—N9—O4	−176.0 (2)
Cd—N3—C10—C9	−1.8 (3)	C41—C42—N9—O4	5.9 (3)
C11—N4—C10—N3	0.3 (2)	C43—C42—N9—O5	3.1 (3)
C19—N4—C10—N3	179.77 (18)	C41—C42—N9—O5	−175.0 (2)
C11—N4—C10—C9	−179.85 (18)	N9—C42—C43—C44	179.53 (18)
C19—N4—C10—C9	−0.3 (3)	C41—C42—C43—C44	−2.4 (3)
O1—C9—C10—N3	3.6 (3)	O9—N11—C46—C45	−128.2 (3)
O1—C9—C10—N4	−176.22 (17)	O8—N11—C46—C45	50.1 (3)
C14—C15—C16—C11	−1.1 (3)	O9—N11—C46—C41	52.6 (4)
C14—C15—C16—N3	178.81 (19)	O8—N11—C46—C41	−129.0 (2)
C10—N3—C16—C11	−1.0 (2)	C43—C42—C41—O3	−177.7 (2)
Cd—N3—C16—C11	−178.40 (13)	N9—C42—C41—O3	0.3 (3)
C10—N3—C16—C15	179.0 (2)	C43—C42—C41—C46	4.1 (3)
Cd—N3—C16—C15	1.6 (3)	N9—C42—C41—C46	−177.99 (18)
C28—O2—C29—C30	161.56 (16)	C45—C46—C41—O3	178.9 (2)
Cd—O2—C29—C30	7.53 (19)	N11—C46—C41—O3	−2.0 (3)
C26—C25—C24—C23	0.4 (3)	C45—C46—C41—C42	−2.7 (3)
C10—N4—C11—C12	178.8 (2)	N11—C46—C41—C42	176.39 (19)
C19—N4—C11—C12	−0.7 (4)	C42—C43—C44—C45	−1.1 (3)
C10—N4—C11—C16	−0.9 (2)	C42—C43—C44—N10	178.50 (19)
C19—N4—C11—C16	179.62 (18)	O6—N10—C44—C43	2.6 (3)
C13—C12—C11—N4	179.7 (2)	O7—N10—C44—C43	−176.9 (2)
C13—C12—C11—C16	−0.7 (3)	O6—N10—C44—C45	−177.8 (2)
C15—C16—C11—N4	−178.85 (17)	O7—N10—C44—C45	2.7 (3)
N3—C16—C11—N4	1.2 (2)	C41—C46—C45—C44	−0.3 (4)
C15—C16—C11—C12	1.5 (3)	N11—C46—C45—C44	−179.4 (2)
N3—C16—C11—C12	−178.50 (19)	C43—C44—C45—C46	2.4 (3)
C30—N8—C31—C32	−179.4 (2)	N10—C44—C45—C46	−177.2 (2)
C39—N8—C31—C32	−7.9 (4)	C58—N16—C56—O18	175.8 (3)
C30—N8—C31—C36	0.6 (2)	C57—N16—C56—O18	1.1 (4)
C39—N8—C31—C36	172.11 (18)	C55—N15—C53—O17	−0.3 (4)
C35—C36—C31—N8	179.79 (19)	C54—N15—C53—O17	178.2 (3)
N7—C36—C31—N8	0.0 (2)	N12—C48—C49—C50	−176.97 (19)
C35—C36—C31—C32	−0.2 (3)	C47—C48—C49—C50	1.2 (3)
N7—C36—C31—C32	−179.98 (19)	C51—C50—C49—C48	−2.8 (3)
C3—C2—C1—N1	−178.4 (2)	N13—C50—C49—C48	175.39 (19)
